# Radioembolization: Is Prophylactic Embolization of Hepaticoenteric Arteries Necessary? A Systematic Review

**DOI:** 10.1007/s00270-016-1310-9

**Published:** 2016-03-02

**Authors:** Alicia S. Borggreve, Anadeijda J. E. M. C. Landman, Coco M. J. Vissers, Charlotte D. De Jong, Marnix G. E. H. Lam, Evelyn M. Monninkhof, Jip F. Prince

**Affiliations:** Division of Radiology and Nuclear Medicine, University Medical Center Utrecht, Heidelberglaan 100, 3584 CX Utrecht, The Netherlands; Julius Center for Health Sciences and Primary Care, University Medical Center Utrecht, Universiteitsweg 100, 3584 CG, Utrecht, The Netherlands

**Keywords:** Radioembolization, Yttrium, Embolization, Gastroduodenal artery, Right gastric artery, Cystic artery, Hepatic falciform artery, Complications

## Abstract

**Purpose:**

To study the effectiveness of prophylactic embolization of hepaticoenteric arteries to prevent gastrointestinal complications during radioembolization.

**Methods:**

A PubMed, Embase and Cochrane literature search was performed. We included studies assessing both a group of patients with and without embolization.

**Results:**

Our search revealed 1401 articles of which title and abstract were screened. Finally, eight studies were included investigating 1237 patients. Of these patients, 456 received embolization of one or more arteries. No difference was seen in the incidence of gastrointestinal complications in patients with prophylactic embolization of the gastroduodenal artery (GDA), right gastric artery (RGA), cystic artery (CA) or hepatic falciform artery (HFA) compared to patients without embolization. Few complications were reported when microspheres were injected distal to the origin of these arteries or when reversed flow of the GDA was present. A high risk of confounding by indication was present because of the non-randomized nature of the included studies.

**Conclusion:**

It is advisable to restrict embolization to those hepaticoenteric arteries that originate distally or close to the injection site of microspheres. There is no conclusive evidence that embolization of hepaticoenteric arteries influences the risk of complications.

## Introduction

Radioembolization has gained widespread usage for the management of both primary and secondary, unresectable and chemotherapy refractory liver malignancies. Because healthy liver parenchyma is mostly supplied by the portal vein, hepatic tumors can be selectively targeted by injection of yttrium-90 (^90^Y) microspheres in the hepatic arteries. Particles of resin or glass, containing millions of the radioactive ^90^Y microspheres, are injected into the liver via the hepatic artery. These microspheres might disperse to surrounding organs through hepaticoenteric arteries, such as the gastroduodenal artery (GDA), right gastric artery (RGA), cystic artery (CA) or hepatic falciform artery (HFA). Non-target embolization might subsequently result in complications, including gastrointestinal ulceration (0.7–28.6 %) [1–4] and cholecystitis (0.6–6.0 %) [5, 6]. Non-target embolization can be prevented through prophylactic embolization of hepaticoenteric arteries during a pretreatment angiography after which technetium-99m-labeled macroaggregated albumin (^99m^Tc-MAA) can be injected as an additional screening procedure.

Experienced centers increasingly omit the occlusion of the vessels originating proximal to the microsphere injection site. Several studies have shown that collateralization and recanalization of arteries can occur after occlusion of hepaticoenteric arteries, opposing the initial purpose of this procedure [7–9] and bringing its benefit into question.

Therefore, the purpose of this review is to evaluate the evidence of prophylactic embolization of hepaticoenteric arteries (i.e. GDA, RGA, CA or HFA) to prevent non-target deposition of microspheres and subsequent complications in patients with liver malignancies undergoing hepatic radioembolization.

## Methods

Reporting of this review was conducted according to the PRISMA guidelines [10].

### Search Strategy

A PubMed, Embase and Cochrane literature search was performed on 22 May 2015 to identify all articles related to the use of embolization of hepaticoenteric arteries in patients with liver malignancies undergoing radioembolization. Search terms used to identify these articles were combinations of ‘liver cancer’, ‘radioembolization’, ‘prophylactic embolization’, all synonyms and MeSH or Emtree terms. After full text screening, references of reviews and identified articles were screened to find additional articles.

### Study Selection

After the removal of duplicates, titles and abstracts were reviewed independently by two reviewers (the first group by A.B. and C.D., the second group by A.L. and C.V.). Full text was obtained if title and abstract met the predetermined in- and exclusion criteria. Disagreements were resolved on consensus-based discussion with all four reviewers. Articles were included in which: (1) patients with liver malignancies undergoing radioembolization were studied, (2) prophylactic embolization of hepaticoenteric arteries was reported, (3) gastrointestinal complications or non-target embolization on imaging was used as outcome, (4) both a group of patients with and without embolization were assessed and (5) the authors reported results in English, German or Dutch. Case reports, animal studies, in vitro studies, congress abstracts and reviews were excluded.

### Risk of Bias

The quality of the studies was assessed by a critical appraisal that was specifically designed for our search and included studies. Studies were independently appraised on validity by four reviewers (A.B., A.L., C.D., C.V.) on the following items: (1) study design characteristics: study type, data collection, funding and potential role of funders in study; (2) standardization: sufficient description of indication for treatment, procedure of embolization, assessment of outcome and (3) loss to follow-up: routine imaging or endoscopy was preferred, but routine clinical assessment was also considered to be of value.

### Data Extraction

Data extraction was performed by two independent reviewers. The following data were extracted from the studies: specification of the embolized arteries, the indication for embolization, study size, number of patients who were embolized or not, results of post-treatment imaging and the number and type of complications in each patient group.

No meta-analysis could be performed due to heterogeneity of the included study populations, the variety of indications used for embolization and the different methodologies used for the assessment of outcomes.

## Results

**Fig. 1:**
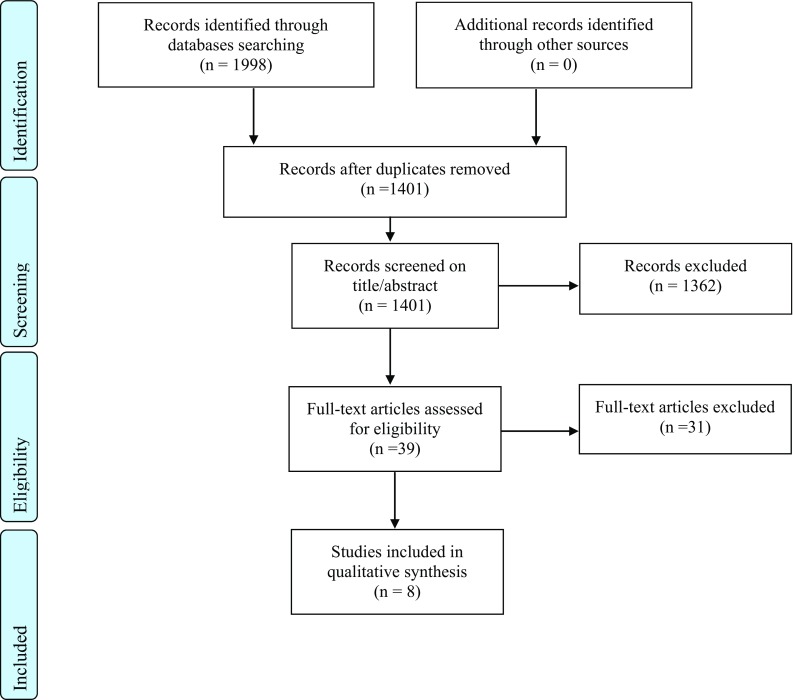
Flow chart of literature search

**Fig. 2:**
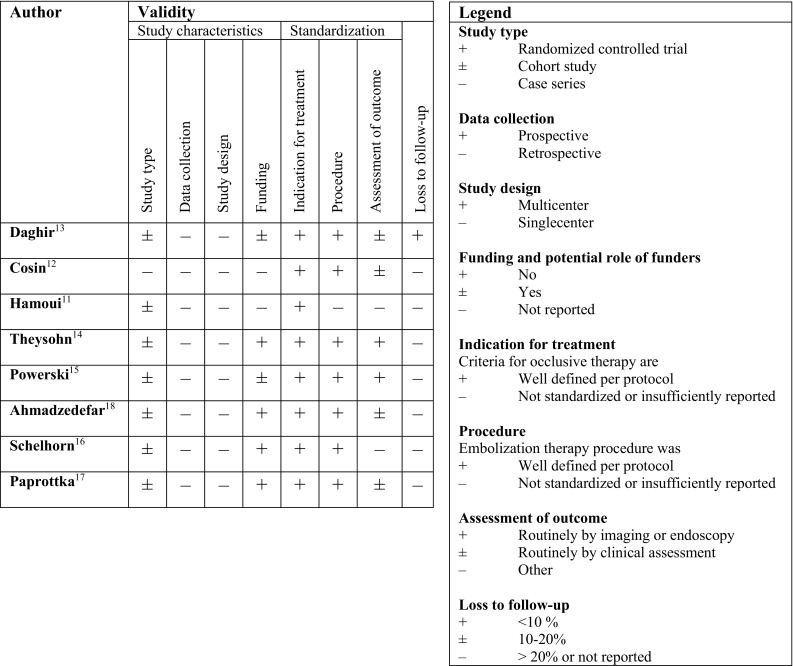
Critical appraisal of selected articles

The search strategy resulted in 1041 articles. Thirty-nine of these articles were screened on full-text. The check for references and related citations did not yield new articles. Eight studies fulfilled the eligibility criteria (Fig. 1) and were assessed for their quality (Fig. 2) [11–18].

The studies were all single-centered, retrospective and non-randomized in nature. There was one letter to the editor [11]. The risk of conflict of interest of all studies was low.

Paprottka et al. [17] and Powerski et al. [15] were considered to be of best quality: both studied a large cohort with even distribution between patients who were embolized or not, and both included a well-defined and extensive follow-up period (respectively 24 weeks and 12 months) (Fig. 2).

Study characteristics and results are listed in Table 1. The included studies investigated a total of 1237 patients of whom 456 received embolization of one or more arteries. Overall, 55 out of 456 embolized patients experienced any type of complication (i.e. adverse events possibly, probably or definitely related to extrahepatic deposition of activity) after radioembolization, varying from pain in the right upper abdominal quadrant to gastrointestinal ulceration. In the non-embolized group, 34 out of 781 patients experienced complications of any kind. The risk differences between patients in the embolized group and patients in the non-embolized group varied from 0 to 12 %.

**Table 1:** Overview of studies comparing complication rates between patients in whom hepaticoenteric arteries were embolized or not

Author	Artery	Indication for embolization	*n*	Embolized *n* (%)	Imaging post treatment	Complications	Not embolized *n* (%)^b^	Imaging post treatment	Complications	Risk difference (95 % CI)^e^
Daghir [13]	GDA	Antegrade flow in GDA and injection position close to GDA. Other arteries, including the RGA, were also embolized	82	71 (87)^c^	*NR*	2 (3 %) duodenal ulceration 1 (1 %) prepyloric ulceration and bleeding	11 (13)	*NR*	0	+3 % (−29 to 11) +1 % (−31 to 9 %)
Cosin [12]	RGA	If visible on angiography and in or close to vascular territory or uptake of ^99m^Tc-MAA	27	9 (33)	*NR*	0	18 (67)	*NR*	0	0 % (−22 to 37 %)
Hamoui^a^ [11]	RGA or GDA	Injection proximal to GDA or RGA	134	42 (31)	*NR*	*NR*	92 (69)	*NR*	2 (1 %) gastrointestinal ulcers^d^	*NA*
Theysohn [14]	Cystic artery	Increased ^99m^Tc-MAA accumulation in the gallbladder wall	295	20 (10)	*NR*	1 (5 %) clinical signs of cholecystitis^d^	275 (93)	*NR*	0	+5 % (0 to 46 %)
Powerski [15]	Cystic artery	If it could be entered swiftly with the wire/catheter	105	68 (65)	3.3 % uptake in gallbladder wall	22 % pain in upper right quadrant 2 (3 %) cholecystitis	37 (35)	8.8 % uptake in gallbladder wall	10 % pain in upper right quadrant 1 (3 %) cholecystitis	+12 % (*NA*) 0 % (−13 to 9 %)
Ahmadzadehfar [18]	Falciform artery	*NR*	17	1 (6)	0	*NR*	16 (94)	9 (56 %) uptake in abdominal wall	1 (6 %) abdominal muscle pain	*NA*
Schelhorn [16]	Falciform artery	If technically possible	11	5 (45)	*NR*	0	6 (55)	*NR*	0	0 % (−48 to 54 %)
Paprottka [17]	*NR*	If catheter could not be placed distally with sufficient safety margin (even if no ^99m^Tc-MAA uptake was present)	566	240 (42)	*NR*	31 (13 %) CTCAE ≥3 within 7 days 3 (1 %) CTCAE ≥3 within 6 months	326 (58)	*NR*	14 (4 %) CTCAE ≥ 3 within 7 days 3 (1 %) CTCAE ≥ 3 within 6 months	+9 % (4 to 14 %) 0 % (−2 to 3 %)

*CI* confidence interval, *RGA* right gastric artery, *GDA* gastroduodenal artery, *NR* not reported, *NA* not applicable

^a^Letter to the editor

^b^Relates to the specific artery, others arteries may be embolized

^c^Not only GDA was embolized, also RGA, cystic and hepatic arteries

^d^Healed after conservative therapy

^e^Risk difference was calculated for the incidence of complications in the embolized group compared to the non-embolized group, i.e. a positive risk difference indicates more complications occurred in the embolized group and vice versa (Wilson procedure with continuity correction)

### Indication for Embolization

The studies were subjected to confounding by indication (the determinant is present if a perceived high risk of poor prognosis is an indication for treatment) [19], because the decision to occlude hepaticoenteric arteries depended on specific clinical situations, e.g. the infusion of microspheres proximal to the origin of the hepaticoenteric arteries [11–18].

All studies gave a detailed description of the pretreatment preparations, the treatment itself and the equipment and materials used, except for Hamoui et al. [11] who specified only the type of microspheres used.

### Assessment of Outcome

Two studies [14, 15] used radiological follow-up, physical examination and blood tests to identify complications due to non-target radioembolization. Theysohn et al. [14] performed a CT-scan of the liver 28 days after radioembolization to detect changes like thickening of the gallbladder wall or free fluid in the gallbladder bed. In the study by Powerski et al. [15], patients received an MRI of the liver on three occasions to assess gallbladder wall thickness and free fluid adjacent to the gallbladder: before pretreatment angiography, immediately after treatment and 6 weeks after treatment. Additionally, bremsstrahlung SPECT was used to detect radioactive microspheres in gallbladder tissue [15].

Five studies used clinical and/or laboratory parameters during follow-up [12, 13, 16–18]. Paprottka et al. [17] was the only study that classified the clinical complications of non-target embolization using standardized criteria, namely the National Cancer Institute’s Common Terminology Criteria for Adverse Events (CTCAEv3.0) [20] and differentiated between early complications (within a week after treatment) and late complications (up to 6 months). Complications of grade ≥3 were considered clinically relevant.

Hamoui et al. did not specify their follow-up procedure, but did seek for histologic evidence of microsphere deposition in patients with gastric ulcers [11].

### Timing of Follow-Up

Follow-up consisted of frequent clinical assessment in five studies [12, 13, 15, 17, 18]: every 2–6 weeks, up to 2 [13], 3 [12], 6 [17] or 12 months [15, 18] after treatment. Other studies did not specify the frequency of post-procedural follow-up visits or instructed patients to contact the hospital in case of complaints [11, 14, 16]. One study [13] mentioned loss to follow-up of patients, but did not take this into account during analysis or interpretation of the data.

### Resin and Glass Microspheres

Two studies [11, 14] used glass microspheres while all other studies used resin microspheres [12, 13, 15–18].

### Gastrointestinal Complications

**Fig. 3:**
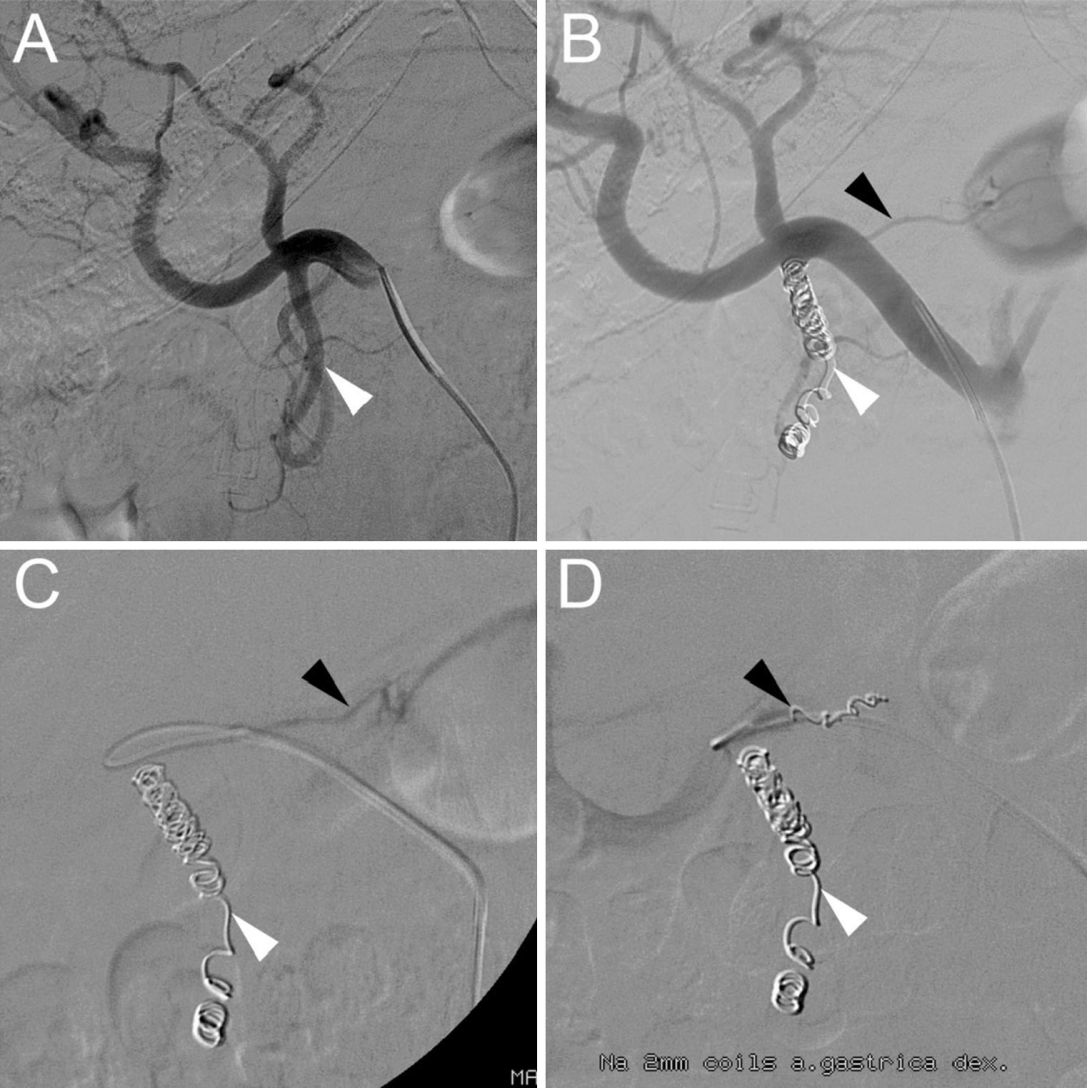
Typical angiography in a patient who underwent coil-embolization of the gastroduodenal artery (GDA) and right gastric artery (RGA). **A** Digital subtraction angiography (DSA) of the GDA (*white arrowhead*) on pre-treatment angiography. **B** DSA with appearance of the RGA (*black arrowhead*) after coil-embolization of the GDA. **C** DSA with catheter placement in the RGA. **D** DSA after successful coil-embolization of the GDA and RGA

Three studies investigated embolization of the GDA or RGA [11–13] when applying specific criteria (Fig. 3). None found a significant difference in the occurrence of complications between the study arms.

The first study, Daghir et al. [13], reported on a cohort of 82 patients in whom the GDA was not embolized if it had reversed (i.e., hepatofugal) flow. None of the 11 patients with reversed flow developed complications related to extrahepatic deposition (gastroduodenal bleeding, ulceration or pancreatitis), but 7 out of those 11 patients experienced early toxicity of the treatment, including liver derangement, radiation hepatitis, anemia, nausea or postembolization syndrome. Within the group of patients with antegrade flow (*n* = 71), two cases of gastroduodenal ulceration and one case of gastroduodenal bleeding were reported. In two of these three cases, a culprit vessel could be found.

The second, Cosin et al. [12], embolized the RGA when it was visible on angiography and close to the injection position (distance not specified). Neither one of the nine embolized nor one of the 18 non-embolized patients showed any complications.

The third, Hamoui et al. [11], posed that injection distal to the GDA or RGA does not require embolization, since the complication rate was low (*n* = 2, 1 % gastrointestinal ulcers). After endoscopic biopsy, microspheres were present in the gastric wall of one patient, but not in the other, who had a history of peptic ulcer disease. The complication rate of the embolized group was not reported.

### Biliary Complications

Three studies [13–15] reported on the need to embolize the cystic artery. In the first, Theysohn et al. [14] embolized patients’ cystic artery if the uptake of ^99m^Tc-MAA in the gallbladder was larger than in the liver and found one complication in the group that was embolized (*n* = 20).

In the second, Powerski et al. [15] performed embolization if the catheter could easily enter the cystic artery. The amount of ^90^Y uptake in the gallbladder wall was lower after embolization, but more patients complained of right upper quadrant pain (22 vs. 10 %). Two patients developed cholecystitis in the embolized subgroup (*n* = 68), and one in the non-embolized subgroup (*n* = 37).

In the third, Daghir et al. [13] mentioned they did not routinely embolize the cystic artery; however, they did not specify the number of patients in whom the cystic artery was embolized or which precautions they undertook to avoid the cystic artery during delivery of the radioembolic material. No signs of gallbladder inflammation or infarction were seen in both patient groups.

### Hepatic Falciform Artery

Embolization of the hepatic falciform artery was evaluated in two small studies [16, 18]. In these studies, the hepatic falciform artery could be identified in only 28 out of 798 patients (3.5 %).

In the first, by Ahmadzadehfar et al. [18], tracer accumulation in the anterior abdominal wall was seen in 17 (9.3 %) patients. The hepatic falciform artery could be identified and embolized in only one patient, who subsequently did not show ^90^Y uptake in the anterior abdominal wall on bremsstrahlung SPECT/CT. Out of the 16 other patients that showed tracer accumulation in the anterior abdominal wall on ^99m^Tc-MAA images prior to radioembolization, only 9 (56 %) showed uptake in the abdominal wall on post-treatment imaging. One of those nine patients developed abdominal muscular pain above the umbilicus. Furthermore, all other hepaticoenteric arteries were also embolized, but the occurrence of complications, other than abdominal muscular pain, was not reported.

In the second, Schelhorn et al. [16] embolized the hepatic falciform artery with coils or gelfoam in a subgroup of five patients. In six patients no embolization was performed, but neither subgroup of patients developed complications. However, unlike Ahmadzadehfar et al. [18], they used ice packs to induce vasoconstriction in the anterior abdominal wall during ^90^Y administration to prevent complications in patients showing a persistently patent HFA that could not be embolized.

### Other

Paprottka et al. [17] embolized all hepaticoenteric branches originating distal to the injection position during the radioembolization procedure. There were significantly less early toxicities (including nausea, vomiting, abdominal pain and fever) in the group without embolization (4 %) compared to the group with embolization (13 %). The milder (grade 1 and 2) complications also occurred significantly less in the group without embolization (35 vs. 60 %).

## Discussion

The purpose of this literature review was to summarize the evidence for prophylactic embolization of hepaticoenteric arteries to prevent complications after radioembolization. We identified eight comparative, non-randomized, retrospective studies. In general, the rate of gastro-intestinal complications after radioembolization was low in both the embolized and non-embolized group. None of the included studies showed evidence in favor of routine performance of prophylactic embolization. However, they did state that when using certain criteria for embolization (Table 1) it appears to be safe to refrain from prophylactic embolization. For example, Paprottka et al. [17] states that coiling might be abandoned if the catheter for applying the microspheres has a distance of at least 2 cm to the first proximal extra-hepatic artery.

The most important limitation of this review is the lack of randomized controlled trials and prospective studies. Embolization of hepaticoenteric arteries was only performed in patients who are at higher risk for complications, which was determined by the hepaticoenteric vascular anatomy. The risk differences that appear to be in favor of non-embolized patients are distorted by confounding by indication, as the study groups are not comparable. Therefore, the evidence is limited and it is only possible to draw conclusions regarding the necessity of prophylactic embolization to decrease the risk of complications in specific situations.

Also, complications may have been underestimated because most studies did not routinely perform follow-up, post-treatment imaging or endoscopy [11–14, 17]. Even though we developed a quality scoring system specifically for this review to assess these kinds of methodological aspects, this may not have captured all the relevant aspects adequately.

Furthermore, the incidence of complications in both embolized and non-embolized patients may partly be explained by the fact that the occurrence of gastrointestinal complications does not only depend on extrahepatic microsphere deposition, but also on patient characteristics such as a history of gastro-intestinal ulcerative disease. One study [11] took histologic evidence of the affected organs into account and could, and thus, proved that the gastro-intestinal complications were attributable to extrahepatic microsphere deposition, rather than other causes. This implicates that the outcome measures used were prone to bias. Furthermore, it is questionable whether symptoms such as pain are truly attributable to non-target radioembolization when they could also be caused by, for example, ischemia of tumor or liver tissue: post-radioembolization syndrome. Misidentification of hepaticoenteric arteries on pre-treatment angiography or the inability to occlude hepaticoenteric arteries due to small size may also have contributed to the incidence of complications in the non-embolized group [11, 13].

Prophylactic embolization might not always be sufficient to prevent non-target deposition of microspheres as four studies reported complications in embolized patients. A possible explanation for this problem is the occurrence of recanalization of embolized arteries, collateral formation or opening of formerly hypoperfused vessels. Several studies report an incidence of recanalization and collateral development of 11–44 % in coiled patients [7–9]. Perhaps timing of prophylactic embolization during the radioembolization procedure itself, rather than during pretreatment angiography, might reduce the incidence of recanalization and collateral development. Of the studies selected in this review, only Paprottka et al. [17] and Theysohn et al. [14] described this approach. Lastly, extrahepatic deposition could occur because of stasis during administration, but the included studies did not investigate this [21].

Compared to glass microspheres, resin micropsheres have a significant embolic effect, which often leads to arterial occlusion, which, in turn, increases the risk of non-target radioembolization through backflow of microspheres [22, 23]. Theoretically, prophylactic embolization might be less important when using glass microspheres, but our study does not provide sufficient data to support this hypothesis. Two studies directly comparing both microspheres show no significant differences in toxicity and survival rates [24, 25].

A significant improvement in the detection of hepaticoenteric shunting can be achieved by using cone beam CT before radioembolization in addition to digital subtraction angiography [26–28]. The potential role and impact of cone beam CT are not assessed in this review, because none of the included studies mentioned the use of cone beam CT for pre-treatment planning. It is expected that using a cone beam CT will reduce the incidence of hepaticoenteric complications.

Future research could provide a higher level of evidence for the criteria to be used for prophylactic embolization. The most important aspect is the comparability of patient groups. There is no need for further studies comparing patients with an indication for embolization to patients without, but there is a need for a study comparing embolization within those groups. For this, a prospective study that uses a pre-defined protocol that defines the indication to embolize is advisable (e.g. minimal distance between catheter tip and hepaticoenteric arteries).

Lastly, the need to embolize hepaticoenteric arteries before radioembolization may be further eliminated in the future since promising results of alternative techniques to prevent non-target deposition of microspheres, such as temporary balloon occlusion and anti-reflux catheters, have been published recently [29–35].

## Conclusion

There is no conclusive evidence supporting prophylactic embolization of hepaticoenteric arteries directly influences the risk of complications. According to the best available evidence, refraining from embolization of the GDA, RGA and CA is justified when the catheter tip can be placed distal to the origin of these arteries or when reversed flow is present in the GDA. The hepatic falciform artery can be embolized if a large uptake in the abdominal wall is seen. Using these criteria, the risk of complications is low.
